# Self-template synthesis of biomass-derived 3D hierarchical N-doped porous carbon for simultaneous determination of dihydroxybenzene isomers

**DOI:** 10.1038/s41598-017-15129-7

**Published:** 2017-11-03

**Authors:** Dejian Chen, Haifeng Zhou, Hao Li, Jie Chen, Shunxing Li, Fengying Zheng

**Affiliations:** 1 College of Chemistry and Environment, Minnan Normal University, Zhangzhou, Fujian, 363000 China; 2 Fujian Province Key Laboratory of Modern Analytical Science and Separation Technology Minnan Normal University, Zhangzhou, Fujian, 363000 China; 3 School of Information and Technology, Northwest University, Xian, Shaanxi 710069 China

## Abstract

Nitrogen doped hierarchical porous carbon materials (HPCs) was achieved by the successful carbonization, using pig lung as biomass precursor. Three-dimensional HPCs constituted with sheets and lines were synergistically inherited from original pig lung. Such structure provided a large specific surface area (958.5 g^−1^ m^2^) and rich porous, effectively supported a large number of electro-active species, and greatly enhanced the mass and electron transfer. High graphitization degree of HPCs resulted in good electrical conductivity. Furthermore, the different electronegativity between nitrogen and carbon atoms in HPCs could affect the electron cloud distribution, polarity and then the electrochemical oxidation kinetics of dihydroxybenzene isomers. Based on these characteristics of HPCs, the electrochemical sensor for dihydroxybenzene isomers exhibited high sensitivity, excellent specificity and stability. Quantitative analysis assays by differential pulse voltammetry (DPV) technology showed that the sensor has wide linear ranges (0.5–320, 0.5–340 and 1–360 μmol L^−1^) and low detection limits (0.078, 0.057 and 0.371 μmol L^−1^) for the catechol, resorcinol and hydroquinone, respectively. This proposed method was successfully applied for simultaneous detection of dihydroxybenzene isomers in river water.

## Introduction

The separation, identification and detection of organic isomers are a great challenge for analytical chemistry due to their similar structures and properties^1^. Catechol (CC), resorcinol (RC) and hydroquinone (HQ) are the isomers of dihydroxybenzene, which generally exists in industrial waste waters, such as that from cosmetic, pesticide, dye and pharmaceutical industries. Those were listed as the priority pollutants for their high toxicity and difficult degradation in the environment^2–4^. Therefore, it is urgent to develop a method for rapid, simple, accurate and simultaneous determination of those dihydroxybenzene isomers.

So far, a number of methods, including chromatography^5,6^, spectrophotometry^7,8^ and pH-based flow-injection analysis^9^, mass spectrometry^10^, fluorescence^11^, and electrochemical techniques^1,12–22^, have been used for dihydroxybenzene isomer detection. In paticular, electrochemical analysis is given more attention, due to its advantages of simple operation, fast response, and low cost. The oxidation peaks of dihydroxybenzene isomers at conventional solid electrodes often overlaps, making it impossible for simultaneous detection. To overcome this critical issue, selective electrochemical determination can be achieved by the electrode modification with nanomaterials (e.g., graphene, carbon nanotubes, gold nanoparticles, and porous carbon)^14,16,20,23^. The electrodes modified with carbon-based nanomaterials for simultaneous determination of dihydroxybenzene isomers have been reported, but the preparation process of these materials is tedious and expensive and the detection limit is unsatisfied^24,25^. Carbon-based nanomaterials with excellent electrochemical performance are thus highly urgent and recently achieved satisfying experimental results^26–30^. Porous carbon with large specific surface area, pore structure, and abundant active sites has aroused widespread interest.

Biomass-derived precursor has been widely studied in recent years for its low cost and diverse microstructure^31^. In addition, heteroatom-doped porous carbon materials with tailorable microstructure, which are very important for high technical products as doping process can greatly improve the electrical and optical features of materials^32–34^, have widely used in the fields of electrocatalysis, adsorption separation, electrochemical energy storage and conversion^35,36^, which can be obtained through the selection of biomass precursor. This method without any chemical agents is very simple and economical compared with other complex synthesis processes. Recent studies have demonstrated that porous carbon has a practical application in electrochemical sensing^37,38^.

**Figure 1:**
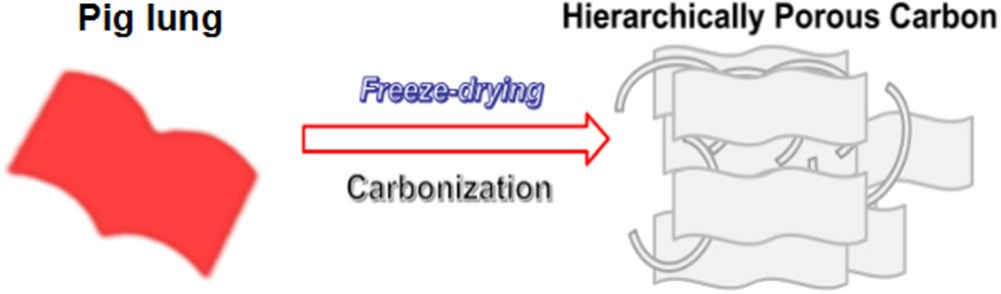
Schematic illustration of the evolution of HPCs.

In this work, fresh pig lung with abundant nitrogen-containing substances is used as the precursor for the first time, and then nitrogen doped hierarchical porous carbon materials (HPCs) is prepared under high temperature under a nitrogen atmosphere (Fig. 1). Because of large specific surface area, abundant pore structure, excellent electrical conductivity, and nitrogen doping, HPCs modified electrode exhibited good electrode conductivity, high electrocatalytic activity, sensitivity and selectivity. When HPCs is used to replace other carbon-based materials for electrode modification, the linear range and detection limit for simultaneous electrochemical determination of dihydroxybenzene isomers are improved.

## Results

**Figure 2:**
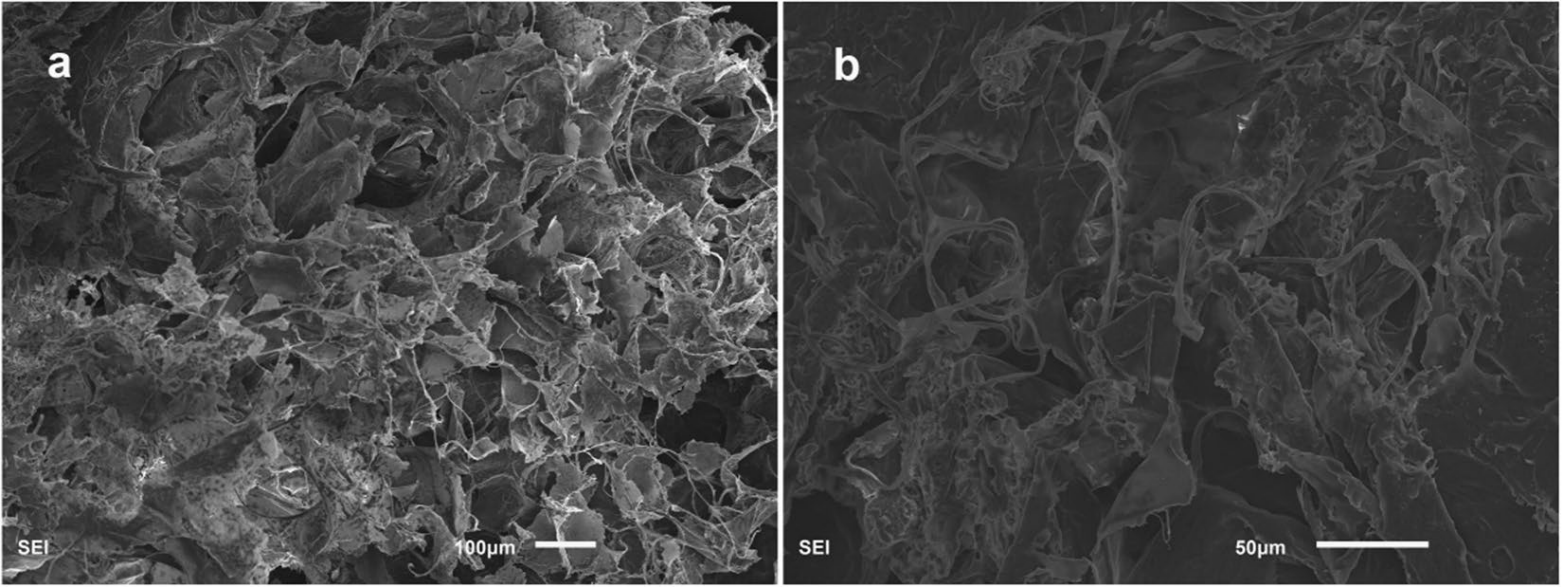
SEM images of pig lung after freeze drying (**a**,**b**).

After characterization by SEM, abundant, three-dimensional, and micron sized holes in dry lung samples were observed in Fig. 2a and b, which were constituted by the lamellae and threadlike. This results proved that the characteristics of the original lung was porous and lightweight, which was an idea material for preparing porous carbon.

**Figure 3:**
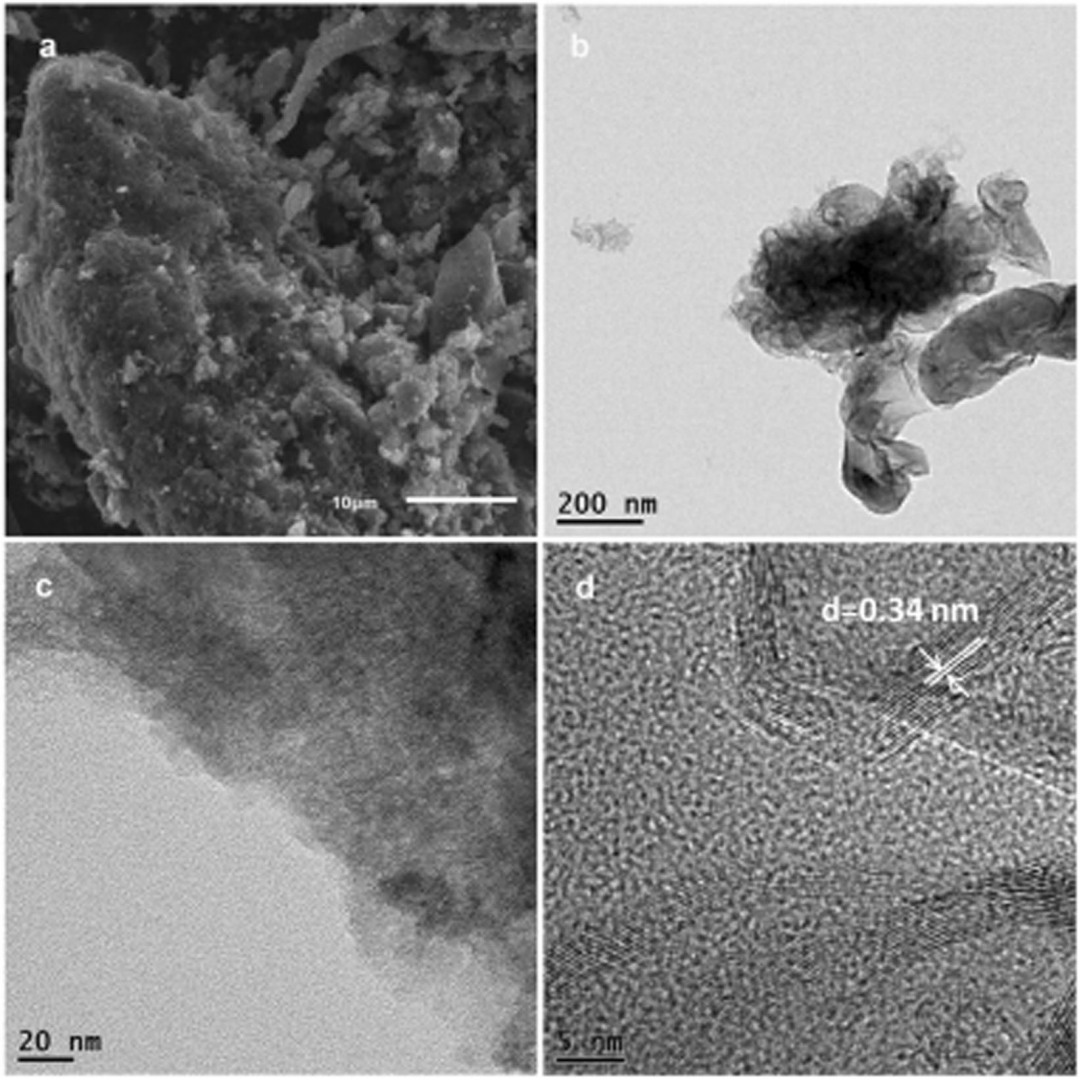
SEM image (**a**) and TEM images (**b**–**d**) of HPCs.

Black powder prepared by pre-carbonize of drying pig lung was characterized by SEM. The pre-carbonization porous carbon materials still retained the lamellar structure with a thickness of about 12 μm, and a lot of porous on the surface of lamellar and the cross section could be seen from the Figure S1a and b, indicated porous carbon materials was formed. In order to obtain carbon materials with higher activity and specific surface area, the pre-carbonization porous carbon materials should be activated. To this end, the pre-carbonization porous carbon materials were further evenly mixed, grinded with potassium hydroxide (1:3, w/w), and then activated under higher temperature. DPV detection of HPCs activated at different calcination temperatures was showed in Figure S2, which indicated that the optimal calcination temperature of HPCs was 800 °C. The obtained HPCs-800 was further characterized by electron microscopy. Figure 3a showed SEM image of HPCs-800, which could be seen that the particles were loose, irregular, rough and porous on the surface. The morphology and structure of HPCs-800 were further characterized by TEM. HPCs-800 was the particles with lamellar structure (Fig. 3b). As shown in Fig. 3c, spongy porous structure in HPCs-800 might lead to a large specific surface area. A similar structure of HPCs prepared by other carbonization temperatures was also observed. A lattice spacing of 0.34 nm was showed clearly by high resolution transmission electron microscopy (HRTEM), which corresponded to 002 crystal plane of graphite. This result indicated that HPCs-800 had a high degree of carbonization. It was widely believed that a good graphitization could exhibit excellent electrical conductivity, so HPCs-800 could provide a good opportunity for the modified electrode detection of dihydroxybenzene isomers.

**Figure 4:**
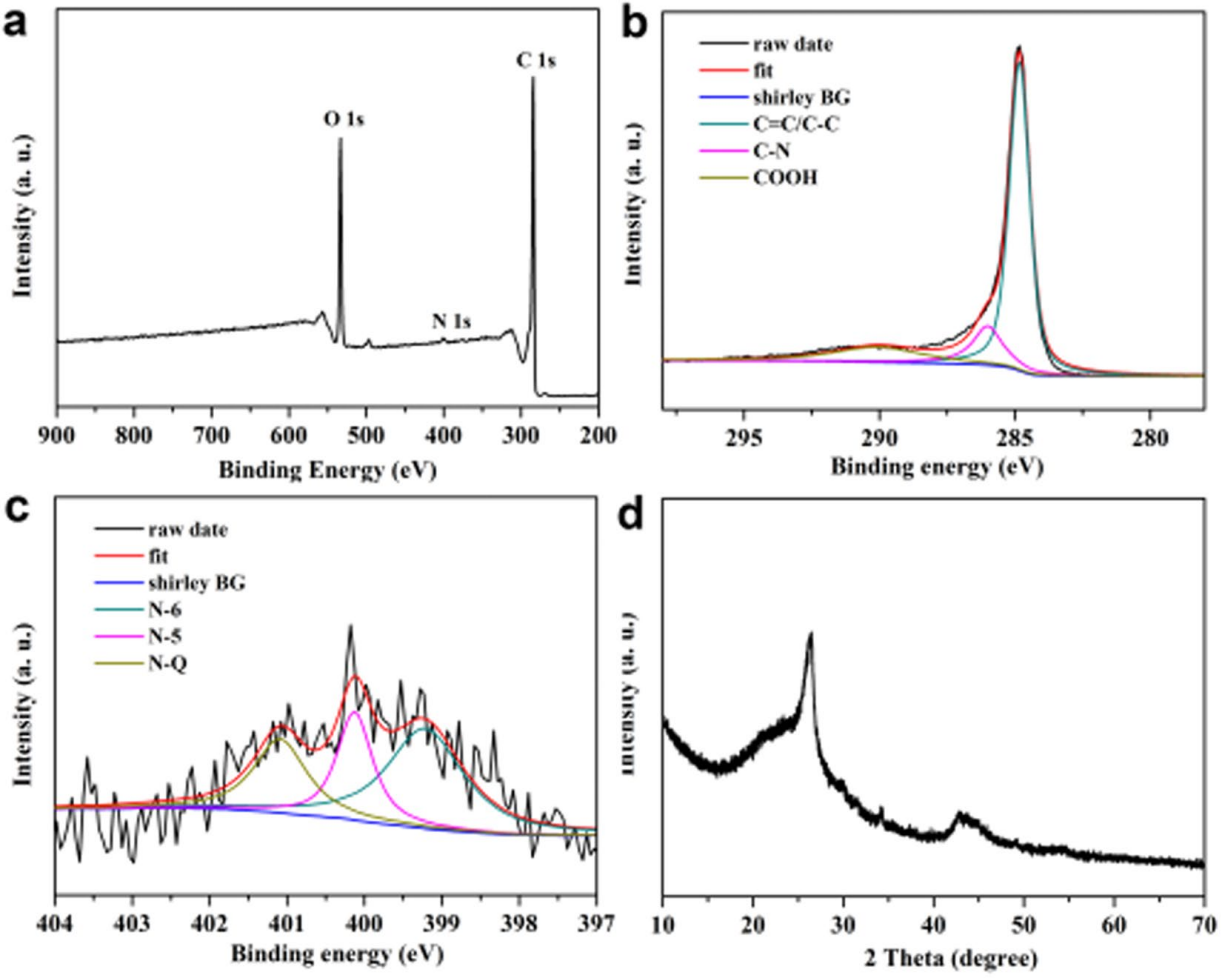
XPS survey spectrum (**a**), C 1 s (**b**), N 1 s (**c**) and XRD patterns (**d**) of as-prepared HPCs-800.

To understand the surface chemistry of HPCs-800, the nature of elemental species was investigated by X-ray Photoelectron Spectroscopy (XPS). The full scan spectrum in Fig. 4a confirmed the formation of N doping in the HPCs-800 and the peaks located at 284.8, 534.05, and 400.12 eV were corresponded to C 1 s of sp^2^ C, O 1 s of the oxygen functional groups and N 1 s of the doped N, respectively. Figure 4b showed the C 1 s spectrum, which could be deconvoluted into three component peaks. The peaks with binding energies at about 284.8, 285.9 and 290.1 eV were attributed to the C-C/C = C, C-N and carboxylic group, respectively. As shown in Fig. 4c, three fitted component peaks centered at 399.2, 400.1 and 401.1 eV in the N 1 s spectrum were ascribed to pyridinic nitrogen (N-6), pyrrolic/pyridone nitrogen (N-5) and quaternary (N-Q) nitrogen, respectively. The XRD pattern of HPCs-800 was showed in Fig. 4d. Two broad peaks at 23.8 and 43.8 were corresponded to the typical multilayer graphite lattice carbon (002) and (100), respectively, which was consistent with the analysis of the HRTEM in Fig. 3d.

**Figure 5:**
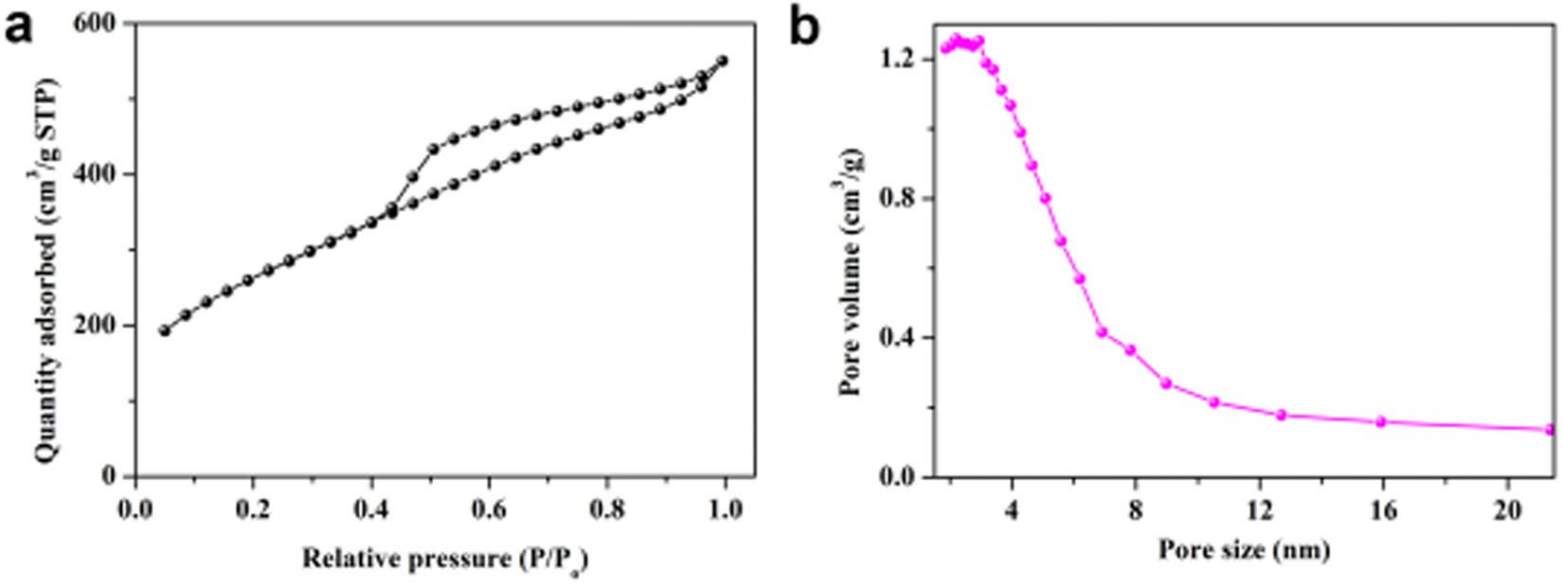
Nitrogen adsorption-desorption isotherms (**a**) and pore distribution curve (**b**) of HPCs-800.

Not only the microscope observation but also the N_2_ adsorption/desorption isotherm were tested to further analyze and evaluate the specific surface area and pore characteristics of the synthesized HPCs-800. For charge storage and conversion, the nanoporous structure of HPCs-800 was highly desirable, because it allowed molecules or ions to quickly pass through and shorten the diffusion pathway. The nitrogen adsorption isotherm of HPCs-800 was presented in Fig. 5a, which exhibited a typical type II/IV curve and a hysteresis loop at relatively low pressure, indicated that the HPCs-800 were micro, mesoporous and macroporous materials^39,40^. Mesoporous structure could be used as an ion transport expressway, while microporous structure helped charge regulation^40–42^. The pore size distribution of HPCs-800 was shown in Fig. 5b, the pore sizes were mainly distributed in the range of 2–4 nm with an average pore size of 3.6 nm and the specific surface area was up to 958.5 g^−1^ m^2^. This large specific surface area of HPCs was helpful to the adsorption and enrichment of target analytes, which might improve the sensitivity of analytical methods.

## Discussion

**Figure 6:**
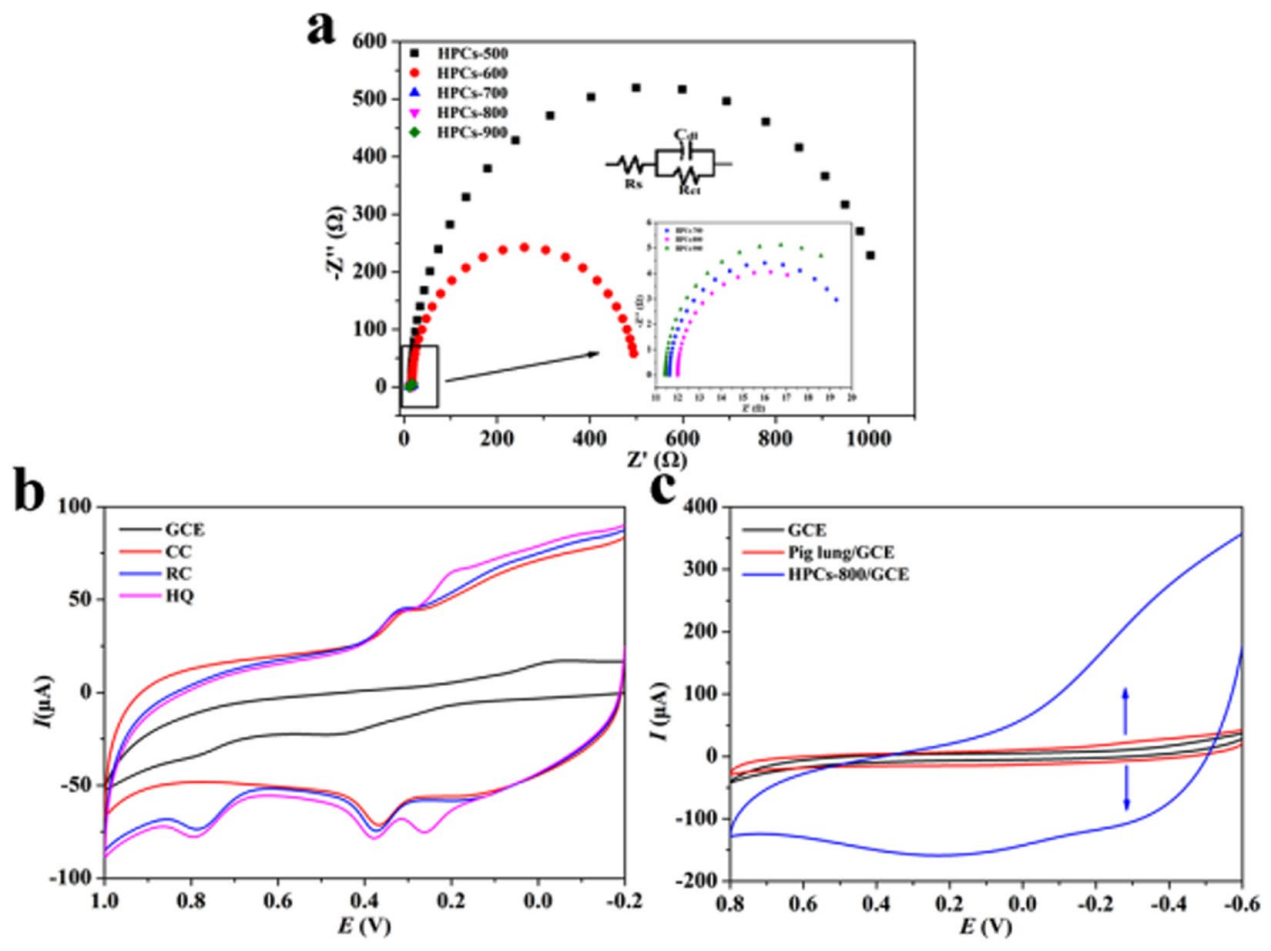
Nyquist plots of HPCs in 1 mmol L^−1^ [Fe(CN)_6_]^3−^/^4−^ solution containing 0.1 mol L^−1^ KCl (**a**), Cyclic voltammetry of bare electrode and HPCs-800 modified electrode in PBS solution (containing 0.1 mmol L^−1^ of dihydroxybenzene isomers) (**b**) and cyclic voltammetry of bare electrode, freeze-dried pig lung and HPCs-800 modified electrode at the scan rate of 100 mVs^−1^ in PBS solution (**c**).

The electrochemical impedance spectroscopy (EIS) was further conducted to study the interface properties of electrode surfaces. The electron transfer kinetics of [Fe(CN)_6_]^3−/4−^ (1 mmol L^−1^, containing 0.1 mol L^−1^ of KCl) on HPCs/GCE calcined at different temperature were shown in Fig. 6a. In the typical Nyquist plot, the diameter of the semicircle part at high frequency was equivalent to the electron transfer resistance (Ret), which controlled the electron transfer kinetics of the redox probe at the electrode interface^43^. The Ret values for different electrodes were in the order of HPCs-500/GCE > HPCs-600/GCE > HPCs-700/GCE > HPCs-900/GCE > HPCs-800/GCE. These results proved the excellent electrical conductivity of HPCs-800/GCE.

In PBS buffer solution (0.1 mol L^−1^, pH 7.0, containing 0.1 mmol L^−1^ of HQ, CC and RC), CV method and bare electrode were used for characterizing their electrochemical behavior. As shown in Fig. 6b, the results showed that the peak current of HQ and CC, RC on bare electrode was very small. The oxidation peaks of HQ and CC were overlaped and only appeared two oxidation peaks. When HPCs-800 modified electrode was used, peak currents were significantly increased, and three oxidation peaks for HQ, CC and RC could be clearly achieved, because of the sensitive electrochemical reaction between HPCs-800 and dihydroxybenzene isomers. Compared the CV spectra of bare electrodes, freeze-dried pig lung modified electrode and HPCs-800 modified electrode at the scan rate of 100 mV s^−1^ (seen in Fig. 6c), the curve of HPCs-800 modified electrode was the widest. Because of large specific surface area and rich porous, HPCs-800 effectively supported a large number of electro-active species, and greatly enhanced the mass and electron transfer. These characteristics were attributed to excellent electronic conductivity because the pig lung was completely carbonized^44^, as well as the electrical charge was accumulated in the double layer mainly by electrostatic forces in the electrode materials of HPCs-800^45^. These active electrons were easy to attract or combine with electroactive substances^46,47^.

The catalytic properties of carbon catalyst are strongly affected by the nature of nitrogen species^44,48^. Pig lung with abundant nitrogen-containing substances was used as the precursor for HPCs. From XPS analysis of HPCs-800, three nitrogen species at 399.2, 400.1 and 401.1 eV in the N 1 s spectrum were ascribed to pyridinic nitrogen, pyrrolic/pyridone nitrogen and quaternary nitrogen, respectively (Fig. 4c)^49,50^. Then, the electronic structure of the carbon atoms located at the edges was affected by the pyridinic nitrogen, which could play a critical role^51^. In particular, nitrogen-doping could disturb the density of state (DOS)^44^ and electroneutrality of graphitic electronic cloud^52^, which could enhance the electron-donor ability and improve their conductivity and catalytic properties^53,54^. Moreover, nitrogen atom with higher electronegativity cause net positive charge for the carbon atoms in HPCs, which could bring good catalytic activity and unique redox properties^55–58^. For those reasons, dihydroxybenzene isomers could be detected on the HPCs-800 modified electrode for the difference of electrochemical oxidation kinetics, which were affected by the electron cloud distribution and polarity for different electronegativity of nitrogen and carbon atoms.

**Figure 7:**
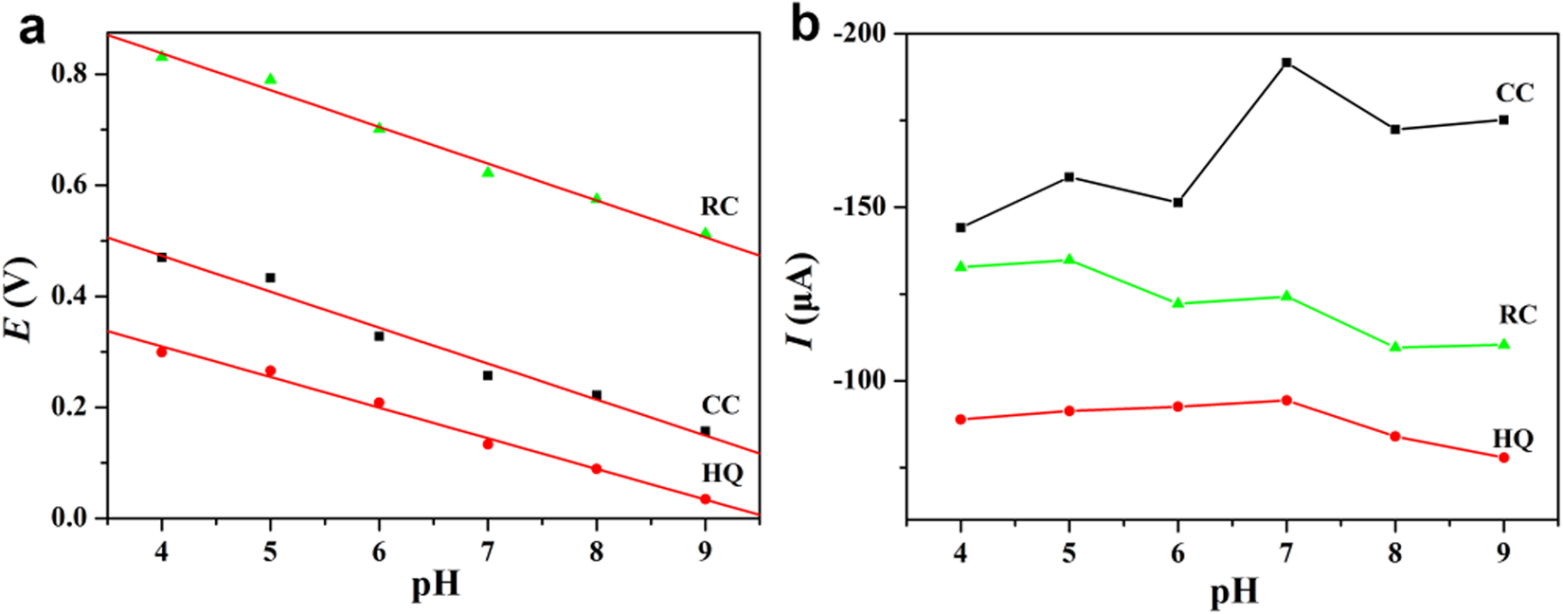
Electrochemical behavior of HPCs-800/GCE in PBS buffer solution with different pH values for 0.1 mmol L^−1^ of HQ, CC and RC. The relationship between E (**a**), I (**b**) and pH.

The effect of buffer pH on the electrooxidation of dihydroxybenzene isomer was also investigated and the results were shown in Fig. 7. The results indicated that the oxidation peak potential of dihydroxybenzene isomer shifted negatively with the increase of the solution pH, which indicated that protons were involved in the electrode reaction. A good linear relationship was established between the oxidation peak potential and the solution pH with the linear regression equation as E_pa_ (V) = −0.05519 pH + 0.53053 (HQ, R = 0.9897), E_pa_(V) = −0.06479 pH + 0.73233 (CC, R = 0.9761), E_pa_(V) = −0.06628 pH + 1.10282 (RC, R = 0.9888). The number of electron transferred could be calculated according to the Laviron equation, *I*_p_ = *n* ^2^ *F* ^2^ *AΓv*(4*RT*)^−1^ = *nFQ v*(4*RT*)^−1^ where *I*_p_ represents the peak current of the anodic or cathodic peak, *Γ* is the surface coverage of the electroactive substance (mol cm^−2^), *A* is the electrode area (cm^2^) and *Q* is the quantity of charge (*C*) calculated from the peak area of the voltammograms, *n* is the number of electron transferred. *F*, *R* and *T* have their usual significance. According to the Laviron equation, the number of electron transferred (*n*) was calculated to be 2.0 approximately, which was the characteristic of a single-electron transfer process. The effect of buffer pH on the oxidation peak current was also investigated and no great differences were found in the pH range from 4.0 to 9.0, so a pH 7.0 PBS solution was selected for all the experiments.

**Figure 8:**
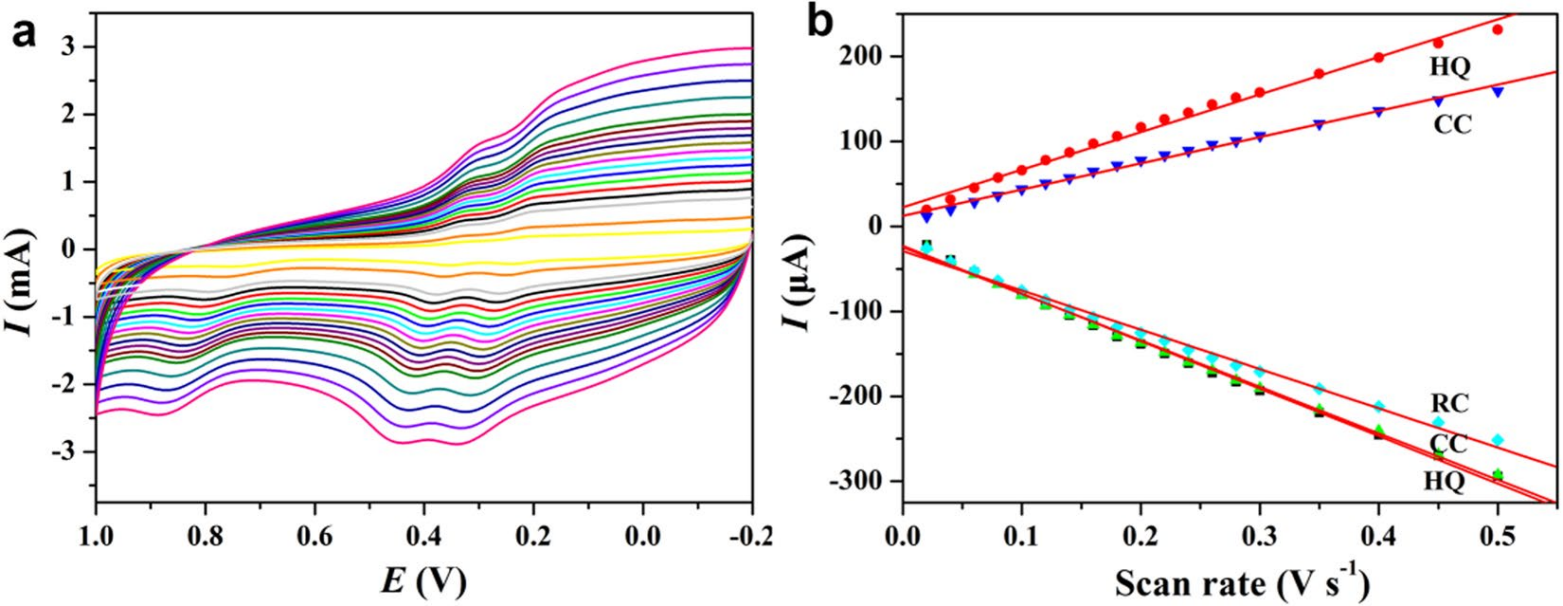
(**a**) Cyclic voltammetry of dihydroxybenzene isomers (0.1 mmol L^−1^) in PBS solution with HPCs-800/GCE at different scan rates (20–500 mV s^−1^). (**b**) oxidation peak and reduction peak current of dihydroxybenzene isomers at different scan rates.

In order to investigate the reaction kinetics of this system, the effect of scan rate on the system was tested. As shown in Fig. 8, the oxidation peaks and the oxidation peaks potential were shifted to the positive potential and negative potential with the increase of the scan rate in the range of 20–500 mV s^−1^, respectively. In addition, the increase of HQ, CC and RC redox peak current, e.g., I_pc_ and I_pa_, was proportional to the scan rate, which indicated that the electrochemical process was a typical adsorption controlled process^59–61^.

The common interfering some inorganic ions and organic compounds (such as Na^+^, Cl^−^, Zn^2+^, SO_4_^2−^, Ca^2+^, Cu^2+^, K^+^, Mg^2+^, NO_3_^−^, phenol, 10-fold concentration of HQ, CC and RC) were added to the mixed solution for evaluating anti-jamming performance of HPCs-800/GCE. The resulted current changes were less than 5%, which indicated that the selectivity and anti-jamming performances was good for HPCs-800/GCE as selective electrochemical sensor for simultaneous detection of dihydroxybenzene isomer. The activity of HPCs-800/GCE for the determination of dihydroxybenzene isomer samples could be kept for at least two weeks, as shown in Figure S3. At the same time, the relative standard deviation (RSD) of the oxidation peak current was 4.85%, which was detected 10 times using same electrode of HPCs-800/GCE. In addition, five HPCs-800 electrodes newly prepared were used for the determination of dihydroxybenzene isomers and the results were shown in Figure S4, which indicated that the reproducibility of this sensor system was excellent. Thus, these results indicated that the HPCs-800/GCE exhibited good stability and repeatability for the detection of dihydroxybenzene isomers.

**Figure 9:**
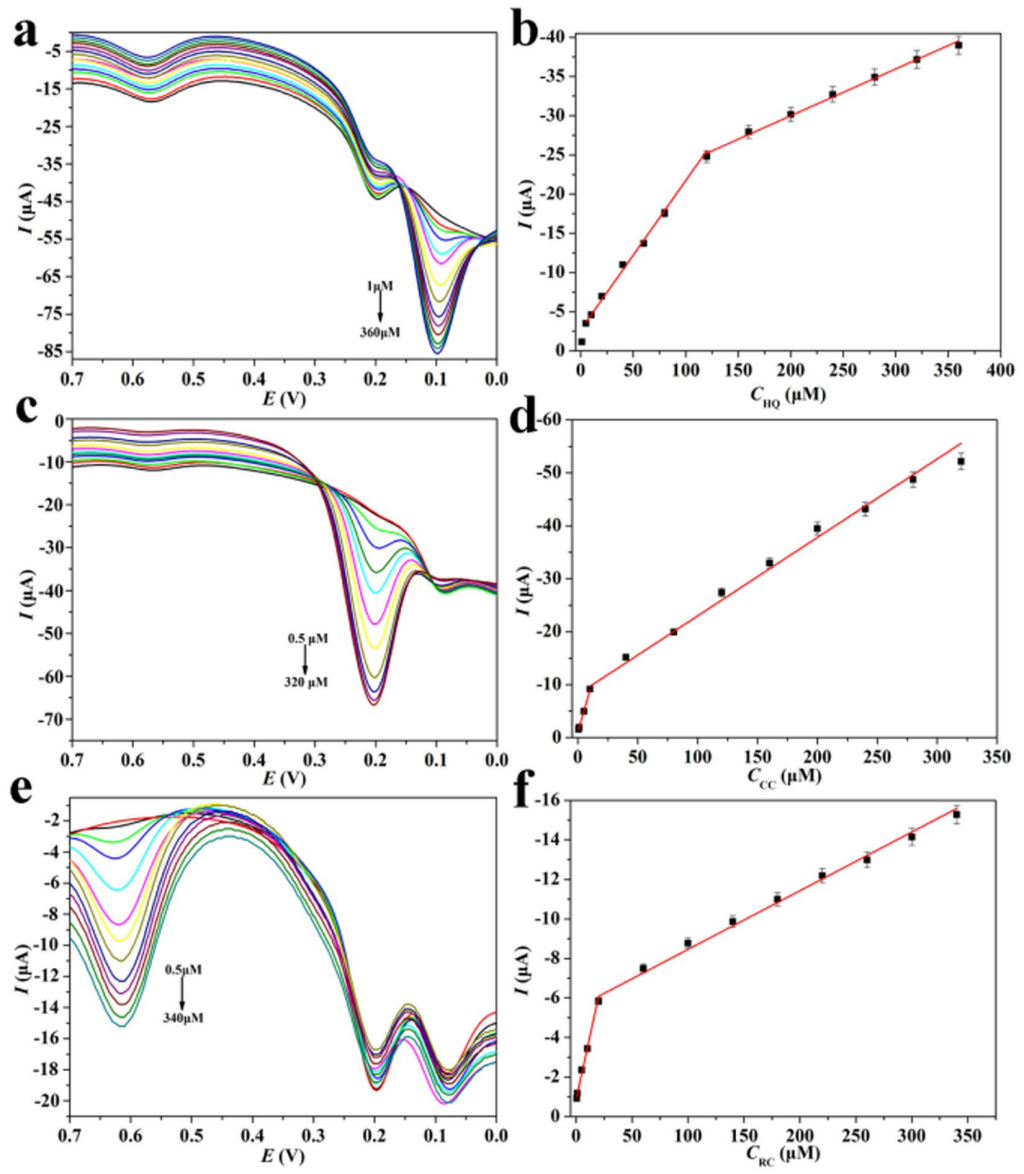
Differential pulse voltammetry for HQ (**a**), CC (**c**) and RC (**e**) in PBS solution with HPCs-800/GCE at the scan rate of 100 mVs^−1^. Calibration plots of oxidation current vs. concentration of HQ (**b**), CC (**d**), and RC (**f**).

Under optimal conditions, we investigated HPCs-800/GCE for simultaneously detecting three kinds of dihydroquinone isomers by DPV technique. For simultaneous and quantitative determination of HQ, CC and RC, DPV spectra at different concentrations of HQ were recorded in Fig. 9a, where CC and RC concentration were kept at 10 mmol L^−1^. With the increasing of the HQ concentration, an anodic peak at the potential of 0.2 V became discernible. The peak current was varied linearly with HQ concentration between 1 and 360 μmol L^−1^ with R = 0.9974. Importantly, the anodic peak current of CC and RC were almost uninfluenced by the increase of HQ concentration, indicated that the oxidations of dihydroquinone isomers at the HPCs-800 modified electrode were independent. Figure S5 showed the simultaneous determination of HQ, CC and RC with HPCs-800/GCE in 0.1 mol L^−1^ of PBS solution (pH 7.0), which further confirmed that HPCs-800 modified electrode could be employed for simultaneous determination of dihydroxybenzene isomers without any interference.

Using DPV technique, the detection limit of HQ was 0.317 μmol L^−1^ (S/N = 3). Similarly, using HPCs-800 modified electrode, wide linear range and low detection limit for CC and RC could be obtained, as shown in Fig. 9c and e, and the calibration plots of oxidation current vs. concentration of HQ, CC and RC could be built in Fig. 9b,d and f. Their linear regression equations were listed in Table 1. The LOD was calculated by the equation LOD = 3 SD/*b*, where SD was the standard deviation of the intercept and *b* was the slope of the regression line, and their linear correlation coefficients were 0.9974, 0.9987, and 0.9967, respectively. At the same time, we found that in the low concentration of dihydroxybenzene isomers on HPCs-800/GCE with response was very sensitive, thus existence an obvious turning point in Fig. 9b,d and f, respectively, which was mainly attributed to the large surface area of HPCs-800 has good adsorption effect to the measured object, thereby greatly improve the detection performance. As an electrochemical sensor for dihydroxybenzene isomers, the advantages of HPCs on the selectivity, sensitivity and detection range were outstanding among the carbon based materials (seen in Table 2).

**Table 1:** Detection of dihydroxybenzene isomer using HPCs/GCE.

Smaple	Linear regression equation I (μA), (μmol L^−1^)	R	Linear range (μmol L^−1^)	LOD (μmol L^−1^)
HQ	I_pa_ = −0.1904c-2.7009	0.9971	1–120	0.317
	I_pa_ = −0.0598c-18.0345	0.9974	120–360	
CC	I_pa_ = −0.7836c-1.1576	0.9987	0.5–10	0.078
	I_pa_ = −0.1480c-8.1984	0.9966	10–320	
RC	I_pa_ = −2.6120c-0.8553	0.9955	0.5–20	0.057
	I_pa_ = −0.0297c-5.4895	0.9968	20–340

**Table 2:** Determination of dihydroxybenzene isomer using carbon based electrode materials.

Electrode	Met.	Liner range (μmol L^−1^)			LOD (μmol L^−1^)			Ref.
		HQ	CC	RC	HQ	CC	RC	
N-CNT/GCE	DPV	10–1000	20–1000	50–1000	1.20	2.70	5.60	24
CNFs/GCE	DPV	1–200	1–200	—	0.20	0.40	—	15
RGO/GCE	DPV	1–500	1–500	—	0.75	0.80	—	62
OMC/GCE	DPV	10–200	10–300	—	0.08	0.10	—	63
GR-GO/GCE	DPV	0.5–300	0.5–300	—	0.16	0.2	—	64
Nafion-FEPA–CNP–GR/GCE	DPV	0.3–90	0.6–100	4–300	0.1	0.2	0.7	25
HPCs/GCE	DPV	1–360	0.5–320	0.5–340	0.32	0.08	0.06	This work

In order to investigate the practicability of modified electrode, HPCs-800/GCE was applied for the determination of isomers in actual water samples under the optimum reaction conditions. Using DPV technique, no target analytes were found in the real water samples (waste water, seawater and river water), indicating that the amount of analytes was below the detection limit. Then, phenol, o-nitrophenol, m-nitrophenol, p-nitrophenol and dihydroxybenzene isomers for their similar structures were added into water samples. No significant interference was observed for the coexistence of other phenolic compounds (Figure S6). Furthermore, according to the standard addition recovery experiment (n = 5), the recoveries were between 98.1% and 104.3%, less than 7%, which demonstrated that HPCs-800/GCE could be used for the detection of dihydroxybenzene isomers in real samples (Table S1).

On the basis of the above results, using fresh pig lung for the self-template synthesis of nitrogen doped hierarchical porous carbon materials was simple, efficient and low-cost. As low density and three-dimensional ordered porous carbon material, the as-obtained HPCs-800 showed good electrical activity and high stability for its large specific surface area and high graphite degree. Moreover, dihydroxybenzene isomers could be simultaneously determined by HPCs-800 modified electrode for the different electronegativity of nitrogen and carbon atoms in HPCs, which could affect the electron cloud distribution, polarity and then the electrochemical oxidation kinetics of dihydroxybenzene isomers. HPCs-800/GCE-based sensing system for dihydroquinone isomers had wider linear range and lower detection limit than other carbon-based materials. The results obtained in actual sample analysis were satisfactory. This method was simple and low cost, providing a good platform for the detection of phenolic compounds.

## Methods

### Preparation of porous carbon

Pig lung was purchased from supermarket (China), cleaned with ultrapure water, and cut into small pieces (1 cm in size), placed in a freeze dryer for 48 h, and then a sponge-like dried pig lung was obtained.

The dried pig lung was pre-carbonized at 450 °C with a heating rate of 5 °C min^−1^ for 3 h in N_2_ flow. The resulting black powder was further grounded and thoroughly mixed with KOH (1:3, w/w) in mortar. Subsequently, HPCs-500, 600, 700, 800 and 900 were prepared by carbonization in a crucible for another 1 h at a temperature of 500, 600, 700, 800 or 900 °C with a heating rate of 5 °C min^−1^ and nitrogen environment, respectively. Finally, the obtained black solid residue was washed with HCl (5%) and abundant ultrapure water, dried at 60 °C for 24 h, and then the porous carbon was obtained. The preparation process of HPCs was shown as Fig. 1.

### Electrochemical Measurements

All electrochemical experiments were carried out on a CHI660E electrochemical workstation (Shanghai Chenhua Instruments Co.) at room temperature. A conventional three-electrode system was used for all electrochemical experiments, which was consisted of a platinum wire, an Ag/AgCl/saturated KCl, and a bare or modified glassy carbon electrode (GCE) as auxiliary, reference, and working electrode, respectively. Prior to the modification, bare GCE were polished with 1.0, 0.3, and 0.05 μm alumina slurry, successively, and sonicated in ultrapure water. The cleaned electrode was dried with high-purity nitrogen steam and modified as follows. The porous carbon (5 mg) was dispersed into dimethylformamide (1 mL) with ultrasonic agitation, the black suspension was dropped onto the GCE surface, dried, thoroughly rinsed with water, and then HPCs-modified GCE was prepared. The cyclic voltammograms were recorded in the range −0.2–1.0 V with sweep speed at 100 mV/s. The initiation and termination potentials of differential pulse voltammetry were −0.2 V and 1.0 V, respectively.

### Instrumentation

The morphology of samples from the preparation process and final products was characterized by scanning electron microscopy (SEM, JSM-6010LA) with an accelerating voltage of 10 kV. Transmissions electron microscopy (TEM) was obtained with Tecnai G2 20 S-TWIN at an accelerating voltage of 200 kV. The TEM samples were prepared by doping solution onto a copper grid and dried in air. The crystal structure of composites was tested by X-ray diffractions analysis master (XRD, D/MAX in Japan-TTRIII). X-ray photoelectron spectroscopy (XPS) was measured by a Thermo ESCALAB 250XI. Raman spectrum was performed by a laser raman spectrometer (Renishaw inVia plus, UK) with 532.05 nm incident radiation and a 50 × aperture. The porous carbon was degassed at 300 °C for 12 h under vacuum and then its nitrogen adsorption isotherm at −196.15 °C was obtained using a Quantachrome, Asic-7 physisorption analyzer. The surface area of samples was evaluated by the Brunauer-Emmett-Teller (BET) model, while the pore size and pore volume were estimated with Barrett-Joyner-Halenda theory.

## Electronic supplementary material

Supplementary Information
