# Supplementary material for: Self-template synthesis of biomass-derived 3D hierarchical N-doped porous carbon for simultaneous determination of dihydroxybenzene isomers
Source: Sci Rep. 2017 Nov 3;7:14985. doi: 10.1038/s41598-017-15129-7 (PMC5670168; doi:10.1038/s41598-017-15129-7)
Supplement: Supplementary file 1 — Supplementary Information [file 41598_2017_15129_MOESM1_ESM.doc]

**Supporting Information**

**Self-template synthesis of biomass-derived 3D hierarchical N-doped porous carbon for simultaneous determination of dihydroxybenzene isomers**

Dejian Chen,^a,‡^ Haifeng Zhou,^a,‡^ Hao Li,^c^ Jie Chen,^a^ Shunxing Li,*^,a,b^ Fengying Zheng^a,b^

*a College of Chemistry and Environment, Minnan Normal University, Zhangzhou, Fujian 363000, China*

*b Fujian Province Key Laboratory of Modern Analytical Science and Separation Technology Minnan Normal University, Zhangzhou, Fujian 363000, China*

*c School of Information and Technology, Northwest University, Xian, Shaanxi 710069, China*

*E-mail:* [*lishunxing@mnnu.edu.cn*](mailto:lishunxing@mnnu.edu.cn)*; shunxing_li@aliyun.com*


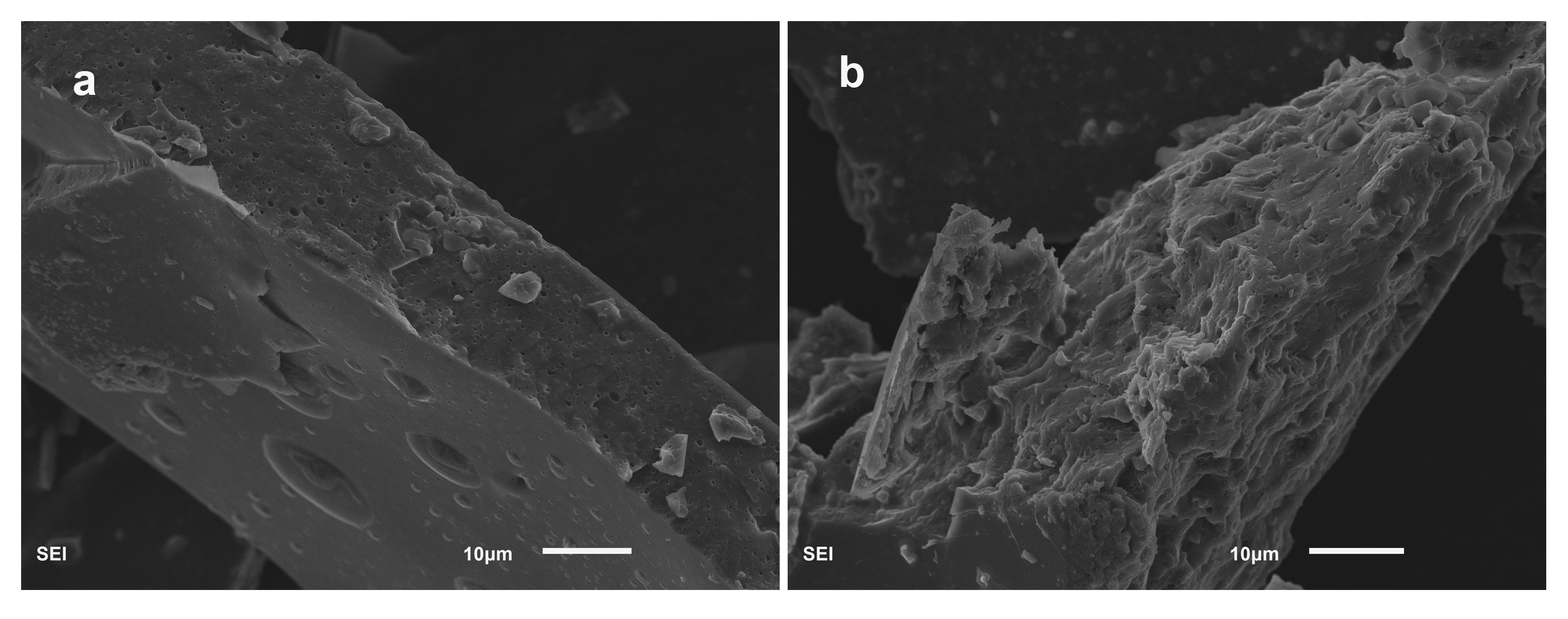


**Figure S1.** SEM images of pre-carbonization porous carbon materials (a and b).


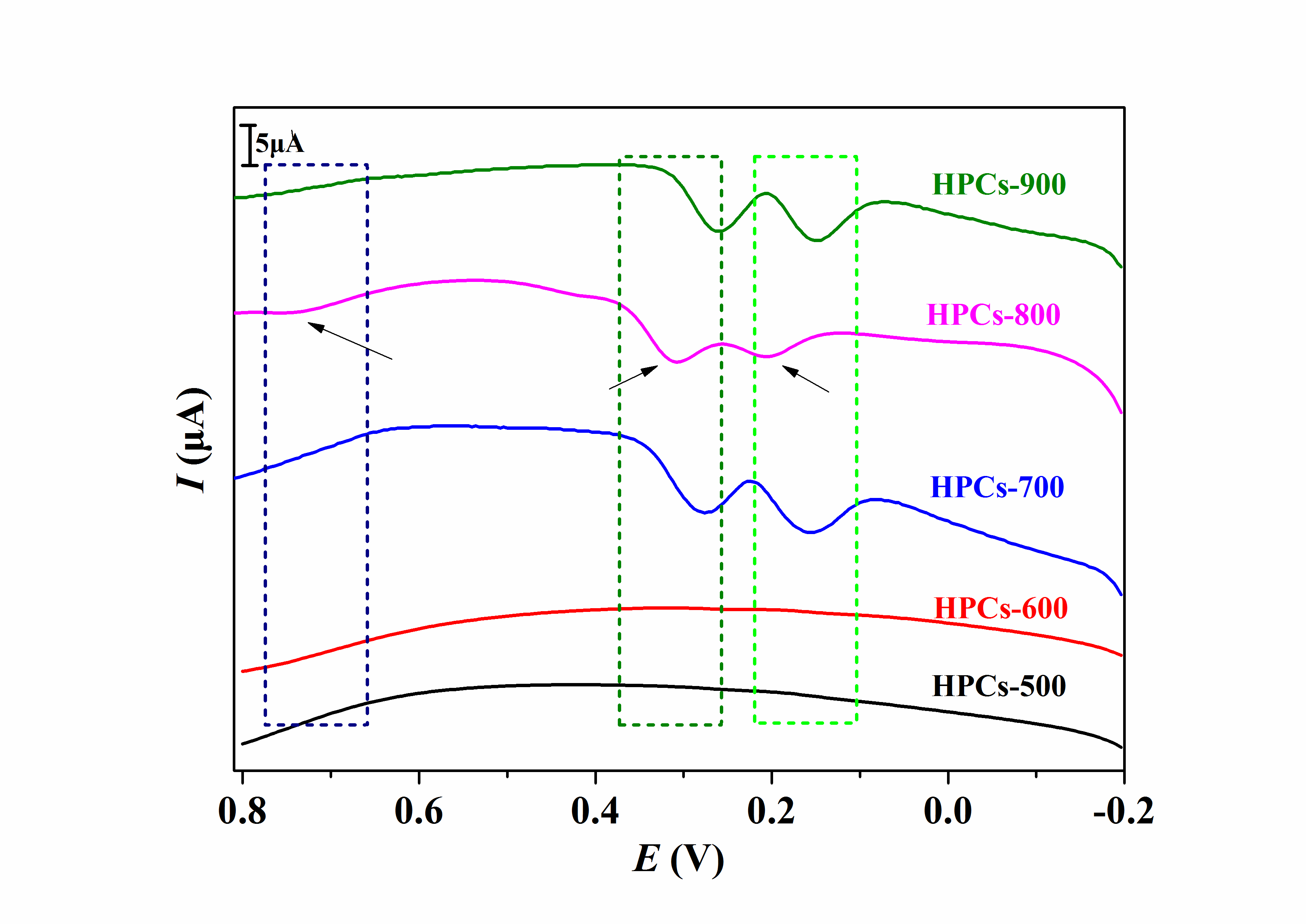


**Figure S2.** DPV detection of HPCs calcined at different temperatures in PBS (0.1mol L^-1^, pH 7.0 ).

Furthermore, Figure S2 showed the results of differential pulse voltammetry (DPV) of HQ, CC, RC (0.1 mmol L^-1^) in PBS (0.1 mol L^-1^, pH 7.0) on HPCs/GCE. With the increase of calcination temperature, the conductivity and oxidation reduction ability were significantly improved, however, RC could not be oxidized by HPCs-700 and HPCs-900, only HPCs-800 modified electrode could simultaneously detected three isomers of dihydroxybenzene. This might due to the structure of HPCs-800 for the presence of graphite, which greatly promoted the electron transfer and thus improved the conductive performance.


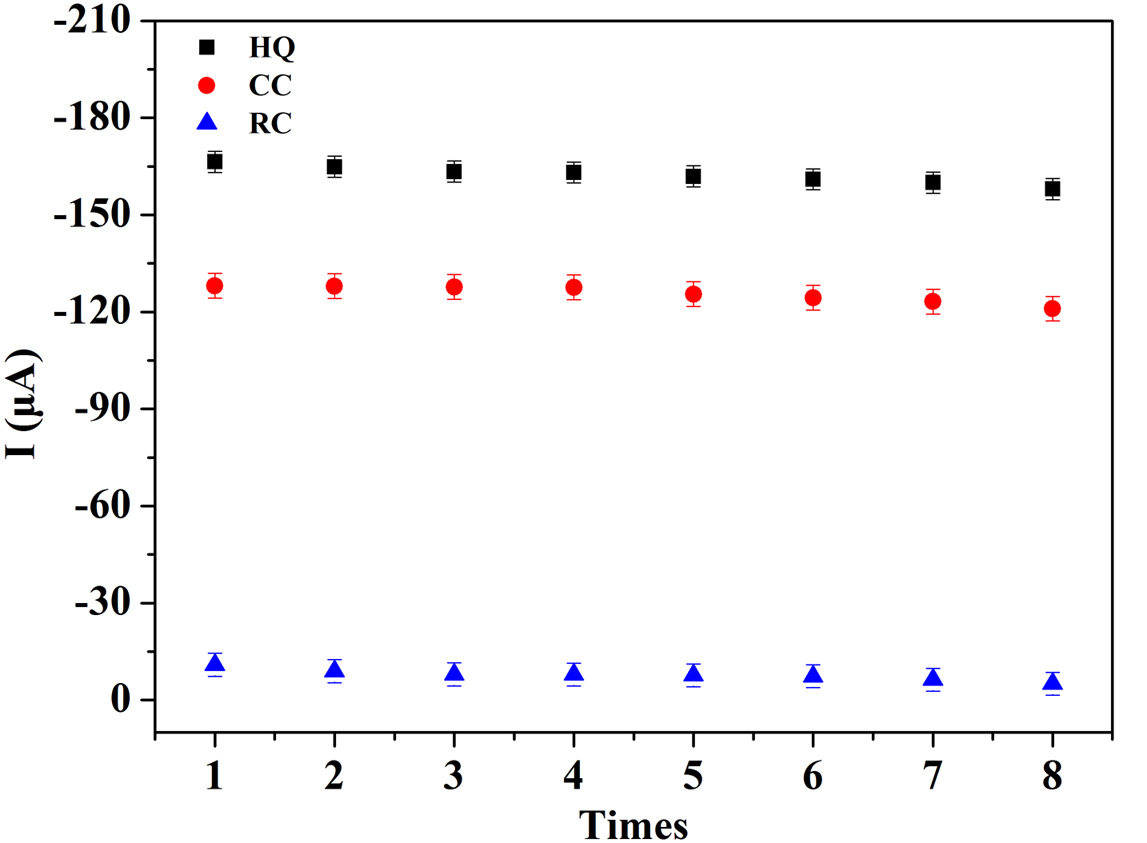


**Figure S3.** Simultaneous determination of dihydroxybenzene isomers 8 times, using HPCs-800/GCE and DPV technology.

As shown in Figure S3, HPCs-800/GCE was used to determine dihydroxybenzene isomer samples 8 times and its activity could be kept for at least two weeks.


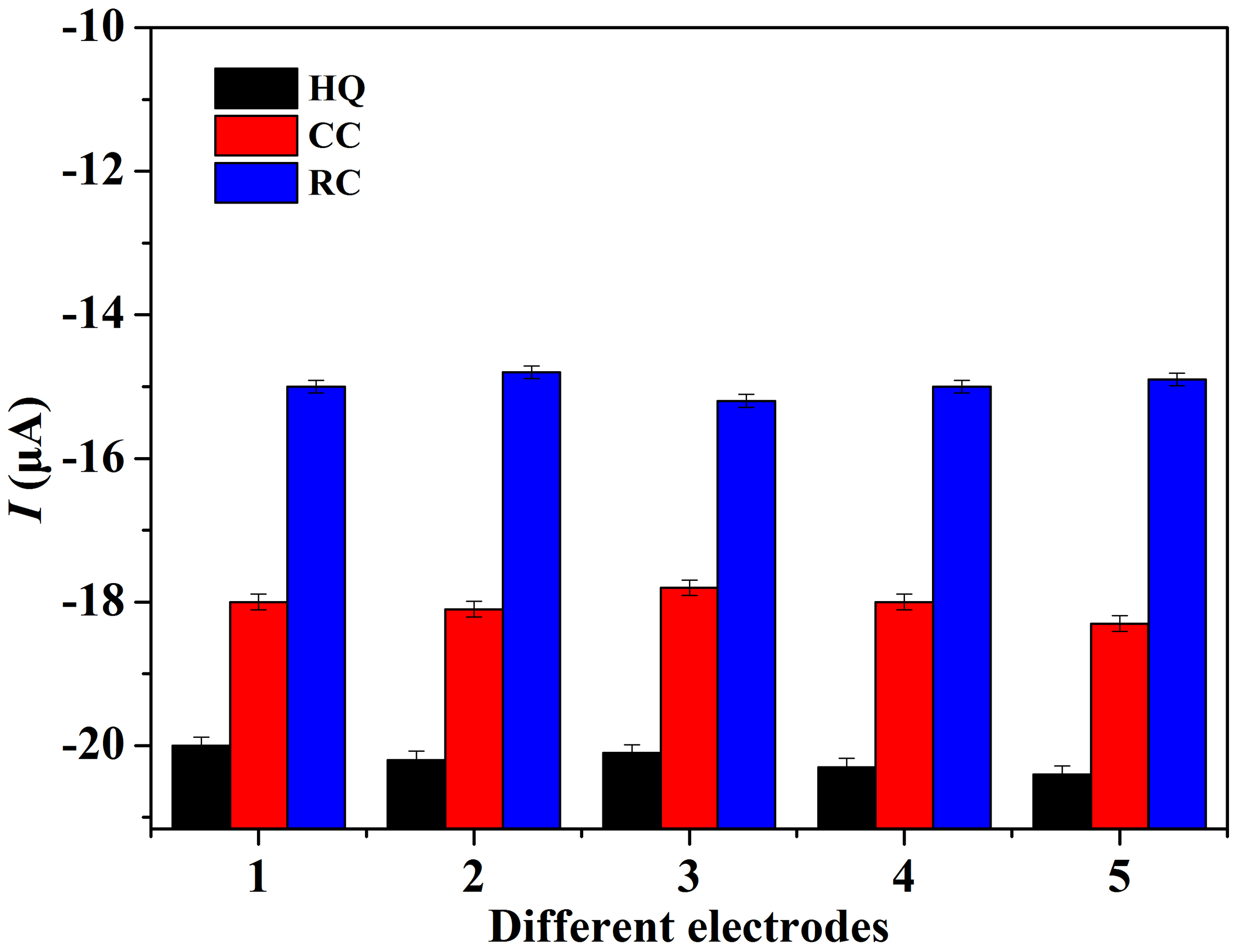


**Figure S4.** Simultaneous determination of dihydroxybenzene isomers by different electrodes.

**
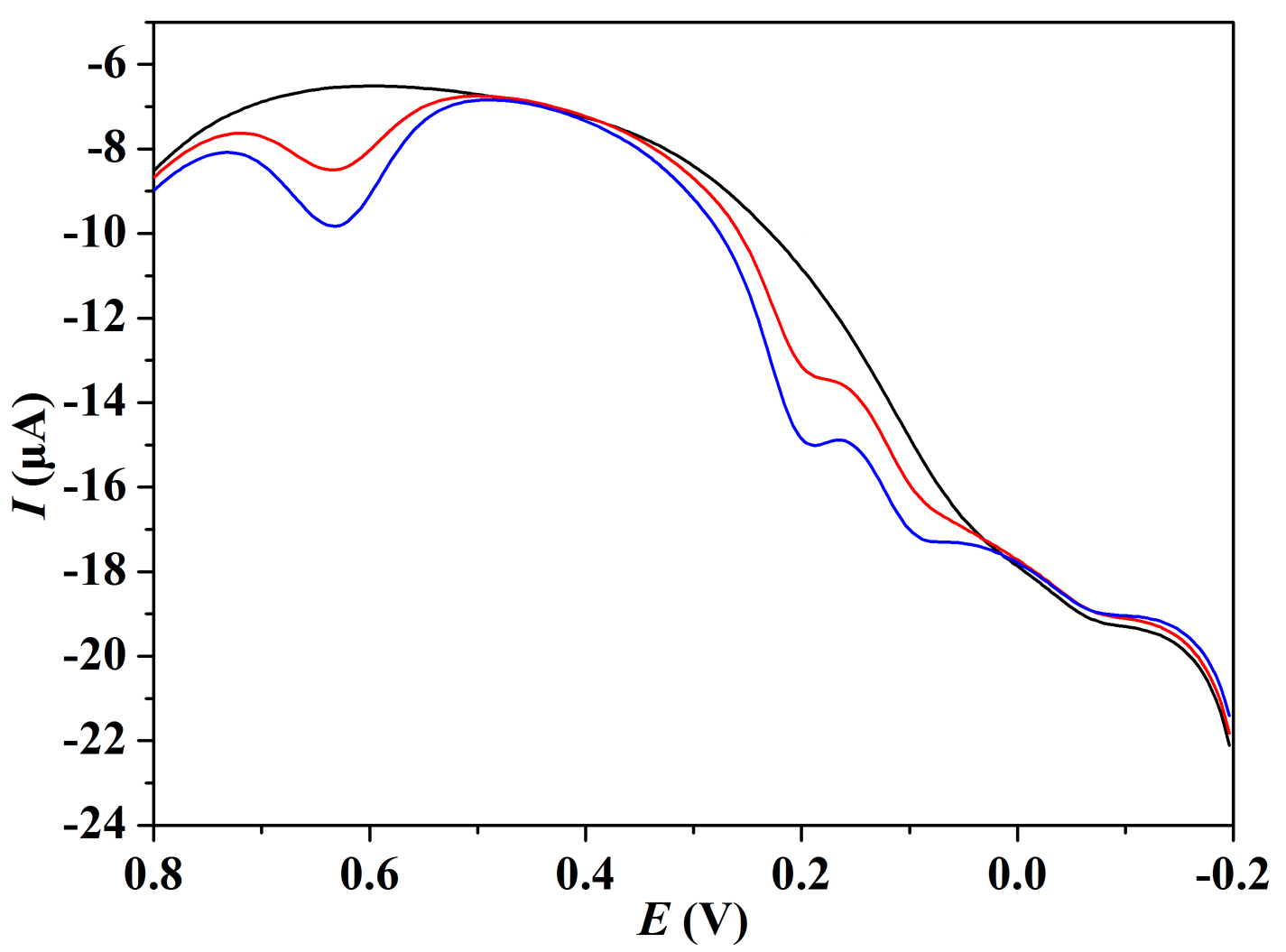
**

**Figure S5.** DPV of simultaneous determination of dihydroxybenzene isomers by HPCs-800/GCE in PBS (0.1 mol L^-1^, pH 7.0).

Figure S5 showed the simultaneous determination of HQ, CC and RC, using HPCs-800/GCE in PBS solution (0.1 mol L^-1^, pH 7.0). The oxidation peak currents for HQ, CC and RC were increased proportionally with the concentration of HQ, CC and RC, ranging from 0, 40, and 60μmol L^-1^, indicating that the HPCs-800/GCE could be used for the simultaneous determination of HQ, CC and RC without any interference.


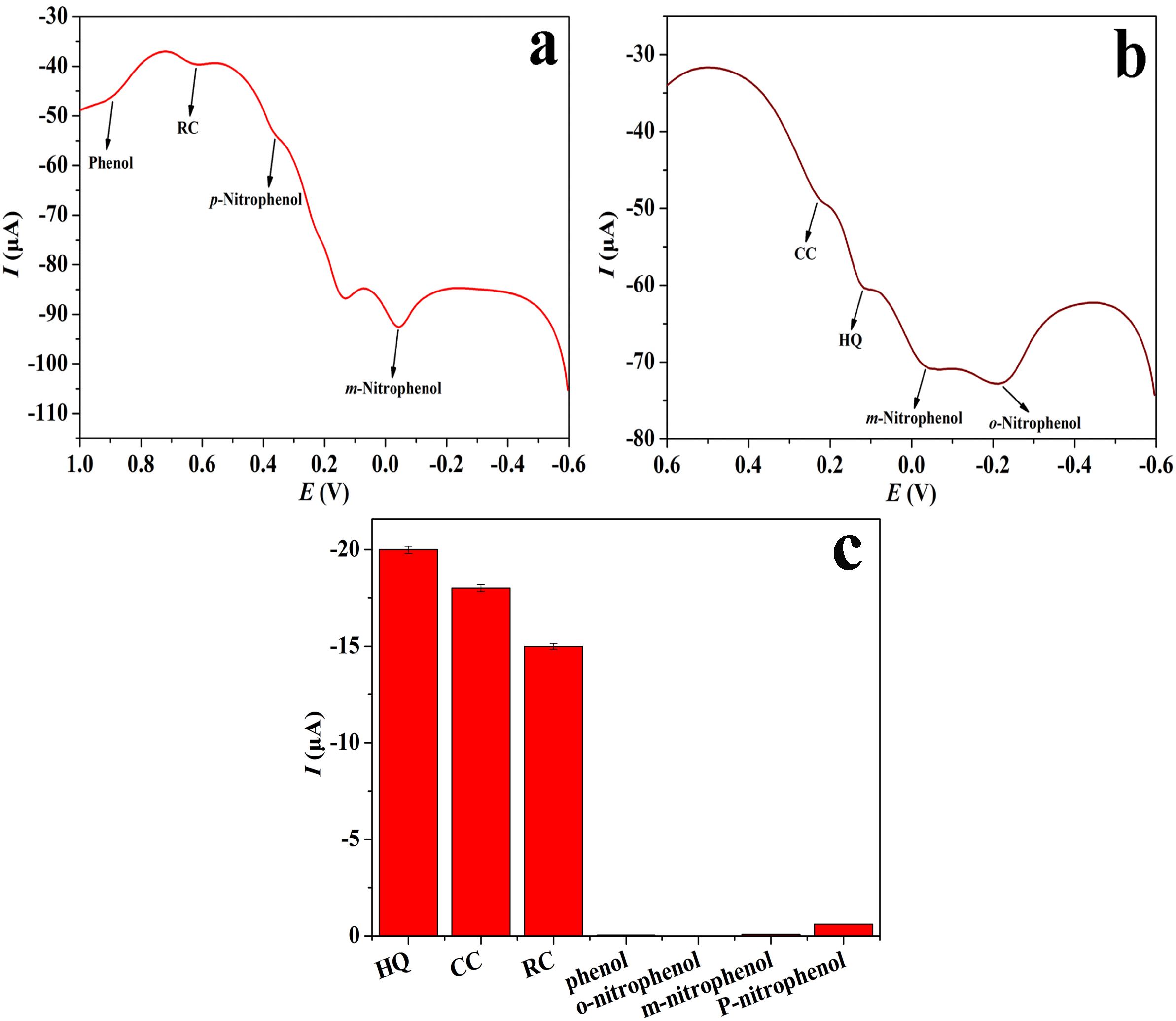


**Figure S6** (a) and (b) DPV of simultaneous determination of 20 μmol L^-1^ dihydroxybenzene isomers by HPCs-800/GCE in river water containing 0.1 mmol L^-1^ phenol, *o*-nitrophenol, *m*-nitrophenol, and *p*-nitrophenol, (c) In the detection of the potential range of 0-0.8 V, the different objects will not produce significant interference.

**Table S1.** Simultaneous determination of dihydroxybenzene isomers in real water samples (ND: no detection)

| **Water Samples** | **Added (μmol L^-1^)** | | | **Found (μmol L^-1^)** | | | **Recovery (%)** | | | **RSD (%, n=5)** | | |
| --- | --- | --- | --- | --- | --- | --- | --- | --- | --- | --- | --- | --- |
|  | **RC** | **HQ** | **CC** | **RC** | **HQ** | **CC** | **RC** | **HQ** | **CC** | **RC** | **HQ** | **CC** |
| Waste water | 0 | 0 | 0 | 0.72 | 0.81 | 0.94 | — | — | — | — | — | — |
|  | 5 | 5 | 5 | 5.63 | 5.77 | 6. 10 | 98.4 | 99.3 | 102.6 | 4.3 | 4.6 | 5,2 |
|  | 10.0 | 10.0 | 10.0 | 10.80 | 10.88 | 10.93 | 101.8 | 104.3 | 103.6 | 4.2 | 5.1 | 5.3 |
| Seawater | 0 | 0 | 0 | ND | ND | ND | — | — | — | — | — | — |
|  | 5 | 5 | 5 | 4.93 | 5.02 | 5.01 | 98.6 | 100.4 | 100.2 | 4.9 | 5.2 | 5.1 |
|  | 10.0 | 10.0 | 10.0 | 9.89 | 9.90 | 9.81 | 98.9 | 99.0 | 98.1 | 5.2 | 6.2 | 4.9 |
| River water | 0 | 0 | 0 | ND | ND | ND | — | — | — | — | — | — |
|  | 5 | 5 | 5 | 4.91 | 4.89 | 5.04 | 98.2 | 97.8 | 100.8 | 5.2 | 5.1 | 6.4 |
|  | 10 | 10 | 10 | 9.96 | 10.04 | 9.89 | 99.6 | 100.4 | 98.9 | 4.3 | 5.2 | 4.2 |
